# Selection of Reference Genes for Expression Analysis in Chinese Medicinal Herb *Huperzia serrata*

**DOI:** 10.3389/fphar.2019.00044

**Published:** 2019-02-01

**Authors:** Mengquan Yang, Shiwen Wu, Wenjing You, Amit Jaisi, Youli Xiao

**Affiliations:** ^1^CAS Key Laboratory of Synthetic Biology, CAS Center for Excellence in Molecular Plant Sciences, Institute of Plant Physiology and Ecology, Shanghai Institutes for Biological Sciences, Chinese Academy of Sciences, Shanghai, China; ^2^University of Chinese Academy of Sciences, Beijing, China; ^3^CAS-JIC Centre of Excellence in Plant and Microbial Sciences, Shanghai, China

**Keywords:** *Huperzia serrata*, lycopodium alkaloids, reference gene, real-time quantitative PCR, gene expression

## Abstract

Huperzine A (HupA) is a powerful and selective inhibitor of acetylcholinesterase. It has attracted widespread attention endangering the ultimate plant sources of Lycopodiaceae family. In this study, we used *Huperzia serrata*, extensively used in Traditional Chinese medicine (TCM), a slow growing vascular plant as the model plant of the *Lycopodiaceae* family to develop and validate the reference genes. We aim to use gene expression platform to understand the gene expression of different tissues and developmental stages of this medicinal herb. Eight candidate reference genes were selected based on RNA-seq data and evaluated with qRT-PCR. The expression of *L/ODC* and cytochrome P450s genes known for their involvement in lycopodium alkaloid biosynthesis, were also studied to validate the selected reference genes. The most stable genes were *TBP, GAPDH*, and their combination (*TBP* + *GAPDH*). We report for the first time the reference gene of *H. serrata’s* different tissues which would provide important insights into understanding their biological functions comparing other *Lycopodiaceae* plants and facilitate a good biopharming approach.

## Introduction

The *Lycopodiaceae* family comprises three main genera, namely, *Huperzia, Phlegmariurus*, and monotypic *Phylloglossum*. The morphological variability between *Phlegmariurus* and *Huperzia* has presented a taxonomic challenge. Interestingly, they possess similar chemical diversity, especially lycopodium alkaloids, such as huperzine A (HupA), a highly potent, selective, and reversible inhibitor of AchE (Zhao and Tang, 2002), hence, a lead candidate for Alzheimer’s disease. HupA was initially isolated from the traditional Chinese medicine Qian Ceng Ta (*Huperzia serrata*). *H. serrata* is an economically important traditional Chinese herb that is used extensively for treatment of contusions, strains, swellings, schizophrenia, myasthenia gavis, and organophosphate poisoning since the Tang Dynasty (Ma et al., 2007; Xu et al., 2017). In the United States, *H. serrata* is marketed as a memory-enhancing dietary supplement (Ma and Gang, 2004). However, the wide clinical investigation and application of HupA are hampered by its poor supply from natural resource or uneconomical synthesis route (Benca, 2014). Moreover, extensive harvest for HupA has endangered *H. serrata* and other species in the Lycopodiaceae family. Synthetic biology approach offers an alternative potential source of HupA, but the inadequate understanding of its biosynthetic pathway restricts its production by metabolic engineering.

**FIGURE 1:**
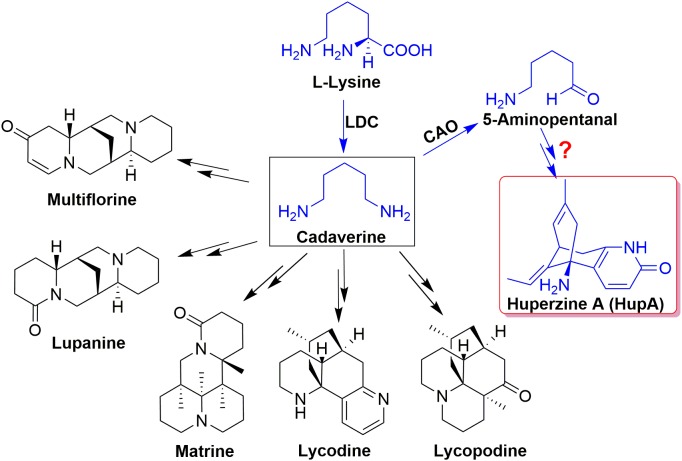
The proposed biosynthesis pathway of Lysine-derived alkaloids, quinolizidine, and lycopodium alkaloids.

Current understanding of the biosynthesis of HupA and other lycopodium alkaloids originates from lysine and/or ornithine from feeding experiments and the pathway was initially proposed lysine/ornithine decarboxylase (*L/ODC*) as the first enzyme (Ma and Gang, 2004). Bunsupa and coauthors reported that *L/ODC* can catalyze the first step in the biosynthesis pathway of lysine-derived alkaloids, quinolizidine, and lycopodium alkaloids (Figure 1; Bunsupa et al., 2012, 2016). Furthermore, we cloned six *HsL/ODC* genes from *H. serrata* by degenerate method and characterized the function of one *HsL/ODC in vitro* and *in vivo* (Xu et al., 2017). A comprehensive relative quantitative metabolomic analysis of these alkaloids in different tissues of *H. serrata* was also performed by our group (Wu et al., 2018). However, the genes involved in skeleton formation and modification remain unclear (Figure 1, blue color; Yang et al., 2017).

Gene expression patterns in different plant tissues and growth developmental stages provide important insights into understanding their biological functions (Bustin, 2000; Vandesompele et al., 2002; Kozera and Rapacz, 2013). Transcriptome analysis and data mining have helped identify differentially expressed genes and measure the relative levels of their transcripts. Quantitative real-time PCR (qRT-PCR) provides a rapid, efficient, accurate, and reproducible method to present the mRNA transcription level in different samples or tissues and to validate data obtained from other methods (Vandesompele et al., 2002; Kozera and Rapacz, 2013; Zhang et al., 2017). The selection and validation of reference genes are the first steps in any qRT-PCR gene expression studies. The most commonly used genes for normalization of gene expression in different plant species include housekeeping genes, glyceraldehyde-3-phosphate dehydrogenase (*GAPDH*), *β-actin, tubulin*, and *18S* (Radonic et al., 2004; Niu et al., 2015; Martins et al., 2017). However, the transcript expression level of such genes is not always stable, especially in samples of different developmental stages and tissues and those subjected to stresses, leading to erroneous results (Radonic et al., 2004; Petriccione et al., 2015; Pombo et al., 2017). Hence, screening and validating reference genes for normalization of the gene expression levels are pivotal.

In this study, *actin, tubulin, 18S, TBP, GAPDH, HSP90, MUB*, and *SAM* were selected as candidate reference genes based on global RNA-seq data. Their expression stabilities in the roots, stems, leaves, and sporangia of *H. serrata* in different developmental stages (2-, 3-, 4-, and 5-year old) were evaluated using geNorm, NormFinder, BestKeeper programs, comparative ΔCq method, and comprehensive stability rankings obtained from RefFinder. The expression of targeted genes, namely, *L/ODC* and cytochrome P450s, which are potentially involved in HupA biosynthesis, were used to validate the selected reference genes. This study is the first report to evaluate the expression stability of the reference genes in *H. serrata*. Results will be particularly useful in the selection of structural genes involved in HupA biosynthesis and research of Lycopodiaceae plants.

## Materials and Methods

### Plant Materials

Plants of different growth periods (2-, 3-, 4-, and 5-year old) were collected from Xiangxi, Hunan, China in January 2017, identified as *H. serrata* (Thunb.) Trevis^1^, and deposited at the Chinese herbarium with Barcode ID: 00019690^2^. The plants were carefully rinsed in running tap water, and soil was removed by hand. Root, stem, leaf, and sporangia were kept in collection tubes immediately after being separated from the plant, immersed in liquid nitrogen, and stored at -80°C until further use.

### RNA Isolation and cDNA Synthesis

Total RNA was extracted from four different tissues of *H. serrata*, namely, root, stem, leaf, and sporangia with TIANGEN RNAprep Pure Plant Kit [Tiangen Biotech (Beijing) Co., Ltd.] according to the kit instructions. DNase I was used to digest contaminated DNA. The purified total RNA was quantified using Nanodrop (Agilent 2100, Agilent Technologies, United States) and 1% agarose gel. cDNA was synthesized as previously reported (Yang et al., 2017).

### Reference Gene Selection and Primer Design

**FIGURE 2:**
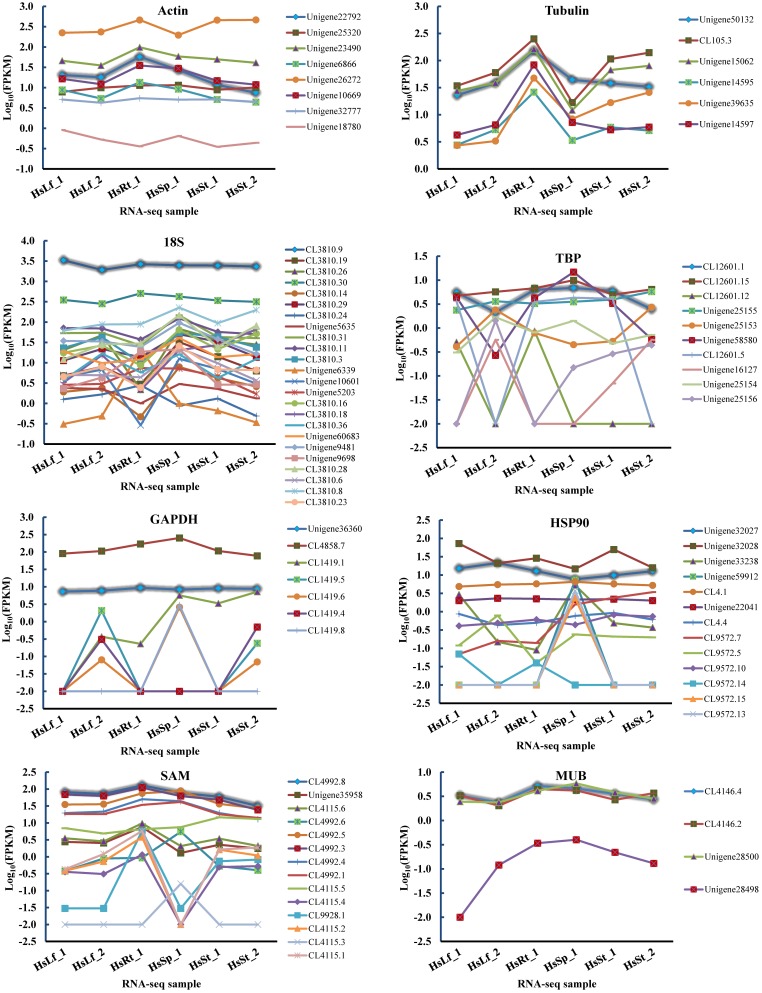
FPKMs of candidate reference genes’ homologs in six RNA-seq samples. The highlight lines indicate the selected candidate reference genes used in this study. Leaf: HsLf_1, HsLf_2; Root: HsRt_1; Sporangia: HsSp_1; Stem: HsSt_1, HsSt_2.

Eight reference genes (*actin, tubulin, 18S, TBP, GAPDH, HSP90, MUB*, and *SAM*) were selected as potential candidates. All homologous in *H. serrata* were gathered by BLAST-search against the global RNA-seq data (Yang et al., 2017), and the candidate reference genes were selected with similar fragments per kilobase per million (FPKM) values determined in the four tissues (Figure 2). The primers of the candidate reference genes were designed, as listed in Table 1. The primer specificities were verified by the presence of a single DNA band with the expected size in 1.0% agarose gel electrophoresis and the presence of a single peak in qRT-PCR melting curve assays (Figure 4).

**Table 1:** Primer sequences and amplification characters for the 8 candidate reference genes.

Gene	Gene ID	Definition	Primer sequence (5′→3′)		R^2^	E
*Actin*	Unigene22792	β-Actin	Actin-F	GCTGTGGTTGTGAAGGAGTATC	0.9989	0.99
			Actin-R	GCTGTGCTGTCTCTGTATGC		
*Tubulin*	Unigene50132	Tubulin	Tubulin-F	AGTCTAGCGTCTGCGATATTG	0.9981	0.97
			Tubulin-R	CCATCTCATCCATACCTTCTCC		
*18S*	CL3810.9	18S ribosomal RNA	18S-F	CAACCATAAACGATGCCGAC	0.9935	1.01
			18S-R	CAGCCTTGCGACCATACTCCC		
*TBP*	CL12601.1	TATA binding protein	TBP-F	CACTGGCTGACTTCCTTCC	0.9987	0.93
			TBP-R	GGCAACTGTTATGTGATTCTCG		
*GAPDH*	Unigene36360	Glyceraldehyde 3-phosphate Dehydrogenase	GAPDH-F	GCCTGCTTCACCACCTTC	0.9960	0.95
			GAPDH-R	GCCTTCCGTGTTCCTACC		
*HSP90*	Unigene32027	Heat shock protein 90	HSP90-F	CTCACTCGCTCCCATTTCC	0.9997	0.92
			HSP90-R	CGCCATCCTCAATCTCTACC		
*MUB*	CL4146.4	membrane-anchored ubiquitin-fold protein	MUB-F	CATCAGAAGGAAGCCATTGTG	0.9983	1.00
			MUB-R	CAGAAGAGCCAGCGTTCG		
*SAM*	CL4992.8	S-Adenosylmethionine decarboxylase	SAM-F	ATGTATTGTAGAATGAGCCTTACC	0.9977	0.95
			SAM-R	CAGCCAAAGAGATGACTAACG		

### qRT-PCR Analysis

qRT-PCR amplification was performed as previously reported (Yang et al., 2017). Expression levels were recorded as cycle quantification (Cq). The PCR efficiency of each primer pair (E = 10^-1/slope^-1) was determined through slope of the amplification curve in the exponential phase, obtained by four fold series dilution of cDNA (Rutledge and Stewart, 2008).

### Gene Expression Stability Analysis

The expression stability of the eight candidate reference genes across all tissues was evaluated with four algorithms, namely, geNorm (Vandesompele et al., 2002), NormFinder (Andersen et al., 2004), BestKeeper (Pfaffl et al., 2004), and ΔCq method. RefFinder (Xie et al., 2012), a web-based user-friendly comprehensive tool, was employed to generate the comprehensive ranking.

### Validation of Identified Reference Genes

Previous studies have shown that *L/ODC* is the first key structure gene for precursor formation of HupA (Bunsupa et al., 2012, 2016; Xu et al., 2017). The homologues of the reported *L/ODC* (Unigene94988, Unigene94617) and four cytochrome P450 genes (CL9415.8, CL1143.2, Unigene1166, and Unigene25121) which were proposed to participate in the modification of HupA skeleton were also used to confirm the reliability of the selected reference genes by using the most two stable versus the least two stable genes.

## Results

### RNA-Seq-Assisted Selection of Candidate Reference Genes and Primer Design

**FIGURE 3:**
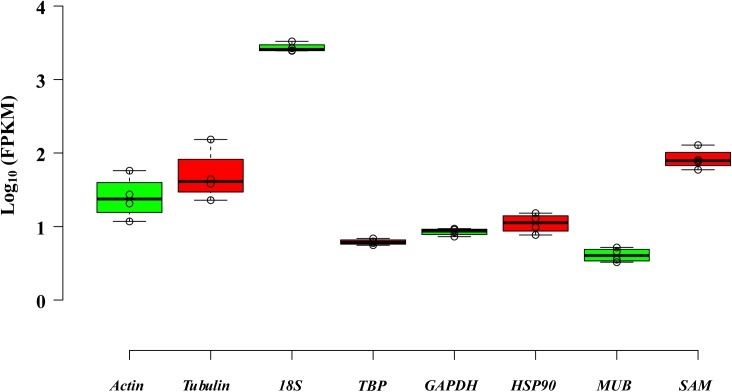
Distribution of eight selected genes by using FPKMs determined in four tissues.

The eight reference genes (*actin, tubulin, 18S, TBP, GAPDH, HSP90, MUB*, and *SAM*) with similar FPKMs in four tissues were gathered, and primers were designed (Table 1, Figure 3, and Supplementary Table S1).

### Expression Levels and Variations of Candidate Reference Genes

**FIGURE 4:**
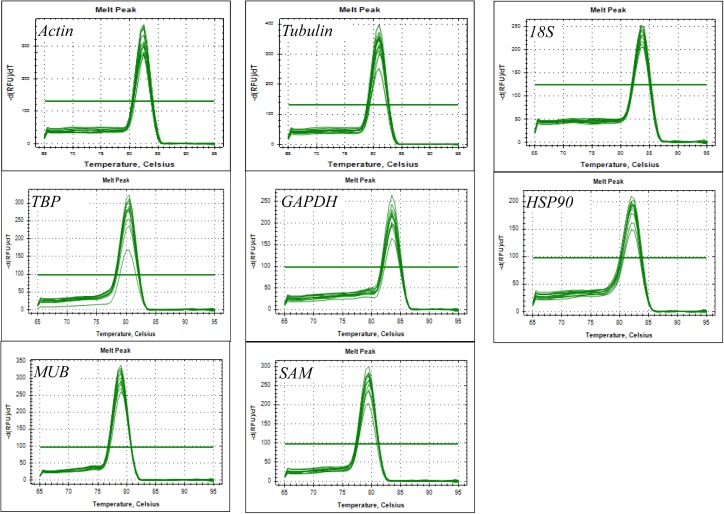
Amplification specificity of primers for qRT-PCR amplification. Melting curves of eight candidate reference genes.

**FIGURE 5:**
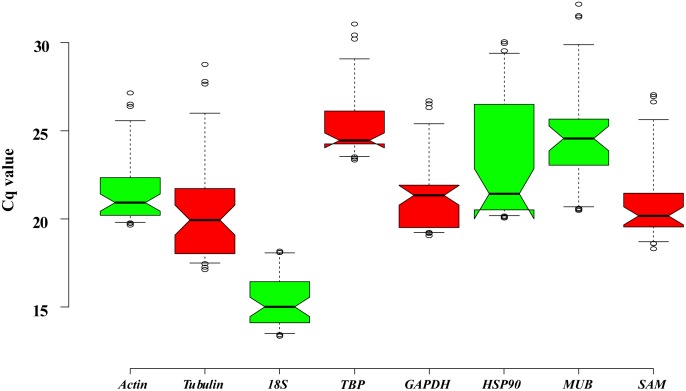
Expression levels of eight candidate reference genes across all experimental samples. Distribution of Cq values of candidate reference genes in all samples. Boxplots show the 25 and 75th percentiles, mean, and outliers.

The PCR product of candidate reference genes were verified by electrophoresis in 1.0 % agarose gel showed only a single band. The presence of a single peak in qRT-PCR melting curve analysis for each of the eight sets of primers indicated high specificity (Figure 4). qRT-PCR was performed to determine the expression levels of each candidate reference genes, and the Cq values showed differential transcript levels in the samples examined with low Cq values, which suggested transcript abundance. The mean Cq value of the eight candidate reference genes ranged from 13.57 to 31.72 (Figure 5 and Supplementary Table S1). In all sample set, the mean Cq values showed a minimum of 15.35 and a maximum of 25.23 for the highest and lowest expression levels for *18S* and *TBP*, respectively. The coefficient of variation (CV) of the Cq values was also calculated to evaluate the expression levels of candidate reference genes of the four tissues, where low values represent low variability or maximum stability. The CV values of the eight reference genes among all samples ranged from 6.91 to 14.60%. *TBP* was the least variable reference gene with a CV of 6.91% among the eight candidate reference genes studied, and *HSP90* was the most variable with a CV of 14.60%. The stability ranking of all candidate reference genes on the basis of CV values is as follows: (most stable to least stable): *HSP90* < *MUB* < *SAM* < *18S* < *GAPDH* < *Actin* < *TBP* (Figure 5).

### Stability of the Reference Gene

Expression stabilities of the eight candidate reference genes were determined using ΔCq, geNorm, NormFinder, and BestKeeper, and their overall stabilities were ranked by RefFinder across all the tissue samples.

### ΔCq Analysis

The eight candidate reference genes from the most to least stable expression, as calculated by the ΔCq method, are listed in Table 2. *GAPDH* and *TBP* were the most stable reference genes in the root and leaf. *Actin* and *TBP* were the most stable genes for the stem and sporangia, respectively. In sum, *TBP, Actin*, and *GAPDH* were the top three ideal reference genes.

**Table 2:** Expression stability of candidate reference genes as calculated by ΔCq.

Rank	Root		Stem		Leaf		Sporangia		Total^∗^	
	Gene	Stability	Gene	Stability	Gene	Stability	Gene	Stability	Gene	Stability
1	*GAPDH*	1.34	*Actin*	1.01	*GAPDH*	1.14	*Actin*	1.34	*TBP*	1.03
2	*TBP*	1.37	*TBP*	1.03	*TBP*	1.15	*TBP*	1.39	*Actin*	1.06
3	*SAM*	1.39	*GAPDH*	1.04	*SAM*	1.33	*SAM*	1.42	*GAPDH*	1.07
4	*Tubulin*	1.48	*Tubulin*	1.18	*18S*	1.35	*GAPDH*	1.51	*SAM*	1.13
5	*Actin*	1.51	*18S*	1.26	*Actin*	1.38	*Tubulin*	1.66	*18S*	1.18
6	*18S*	1.81	*SAM*	1.27	*Tubulin*	1.38	*18S*	2.74	*Tubulin*	1.22
7	*MUB*	2.68	*MUB*	2.37	*MUB*	2.57	*HSP90*	2.92	*MUB*	2.63
8	*HSP90*	4.43	*HSP90*	3.08	*HSP90*	3.52	*MUB*	3.13	*HSP90*	3.44

*^∗^ Total: Pooled samples from all treatments.*

### geNorm Analysis

**FIGURE 6:**
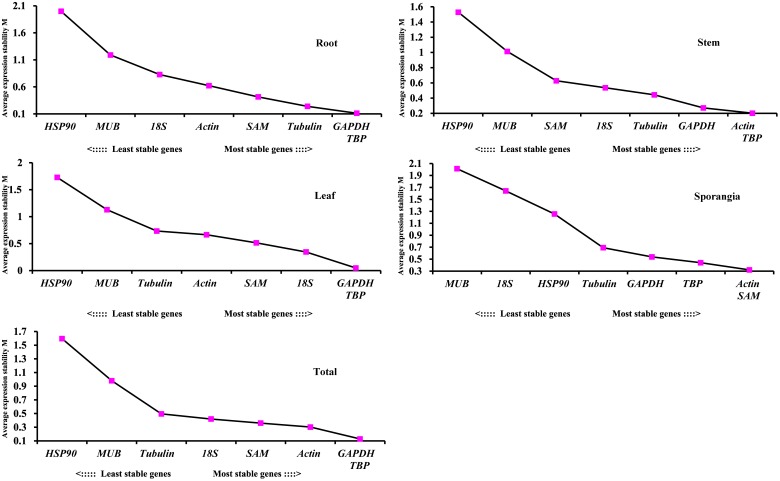
Average expression stability values (M) of the eight candidate reference genes assayed with geNorm software.

The stabilities of the eight candidate reference genes of *H. serrata* calculated using geNorm were ranked in the different tissues according to their M values, as shown in Figure 6. The lowest M value indicates the most stable reference gene, and the highest M value indicates the least stable one. Using the criteria of M < 0.5, *TBP* and *GAPDH* were stable reference genes in the four tissues of root, stem, leaf, and sporangia. When the stabilities from all the samples were combined, *TBP* and *GAPDH* were also determined to be the most stable reference genes. By contrast, *HSP90* and *MUB* were two common unstable reference genes in all tissues and developmental stages.

**FIGURE 7:**
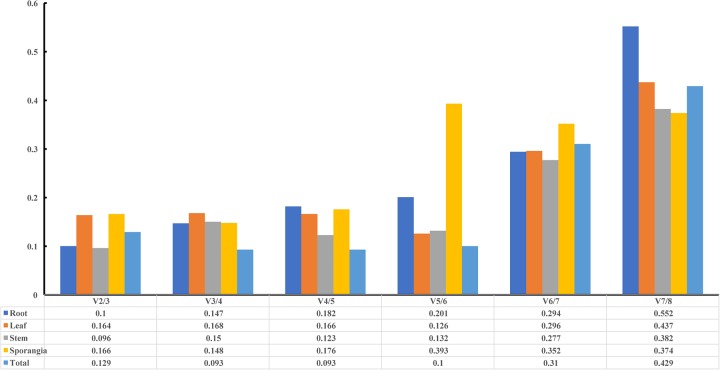
Pairwise variation (V) of candidate reference genes, as calculated by geNorm software. Vn/Vn+1 values were used to determine the optimal number of reference genes.

The pairwise variation (V_n_/V_n+1_) between two sequential normalization factors NF_n_ and NF_n+1_ was calculated by the geNorm algorithm to determine the optimal number of reference genes for accurate normalization. A cutoff value of 0.15 is the recommended threshold, which indicates that an additional reference gene will inconsiderably contribute to the normalization. The V3/4 values in the root and stem were less than 0.15 (Figure 7), which suggested that the top two reference genes were sufficient for accurate normalization. For the leaf, V5/6 was 0.126, which indicated that the top five reference genes (*TBP, GAPDH, 18S, SAM*, and *actin*) were needed for accurate normalization. For the sporangia, V3/4 was 0.148, which showed that three reference genes (*actin, SAM*, and *TBP*) were required. The value V2/3 for total was 0.129, which indicated that the most stable genes, *TBP* and *GAPDH*, could be used as the reference genes for the normalization of gene expression in *H. serrata*.

### NormFinder Analysis

As shown in Table 3, *TBP* and *GAPDH* were the most stable genes (lowest stability value) in the root, leaf, and total subsets calculated using NormFinder. For the stem and sporangia samples, *actin* and *TBP* were the most stable reference genes. When all samples were taken to determine the stability of reference genes, the two most stable genes were *TBP* and *GAPDH*. *SAM* and *actin* also had low stability values, which indicated that the two reference genes were also suitable for qRT-PCR normalization, although not the most stable candidates.

**Table 3:** Stability analysis of candidate reference genes, as assayed with NormFinder software.

Rank	Root		Stem		Leaf		Sporangia		Total^∗^	
	Gene	Stability	Gene	Stability	Gene	Stability	Gene	Stability	Gene	Stability
1	*TBP*	0.005	*Actin*	0.006	*TBP*	0.001	*Actin*	0.006	*TBP*	0.005
2	*GAPDH*	0.005	*TBP*	0.006	*GAPDH*	0.001	*TBP*	0.006	*GAPDH*	0.005
3	*SAM*	0.008	*GAPDH*	0.007	*SAM*	0.020	*SAM*	0.007	*SAM*	0.007
4	*Tubulin*	0.014	*Tubulin*	0.024	*Actin*	0.023	*GAPDH*	0.028	*Actin*	0.007
5	*Actin*	0.025	*SAM*	0.027	*18S*	0.026	*Tubulin*	0.032	*18S*	0.014
6	*18S*	0.073	*18S*	0.049	*Tubulin*	0.034	*18S*	0.098	*Tubulin*	0.015
7	*MUB*	0.107	*MUB*	0.101	*MUB*	0.108	*HSP90*	0.106	*MUB*	0.107
8	*HSP90*	0.175	*HSP90*	0.132	*HSP90*	0.145	*MUB*	0.113	*HSP90*	0.139

*^∗^ Total: Pooled samples from all treatments.*

### BestKeeper Analysis

BestKeeper determined the stabilities of the candidate reference genes on the basis of their standard deviation (SD). Genes with SD>1 were considered unacceptable reference genes. The genes are listed from most to least stability in Table 4. Actin was the most stable gene in the root and total subsets, *GAPDH* was the most stable genes in the stem and leaf subsets, and *18S* was the most stable gene in the sporangia. Only *MUB* and *HSP90* were unstable genes.

**Table 4:** Stability analysis of candidate reference genes, as assayed with BestKeeper software.

Rank	Root			Stem			Leaf			Sporangia			Total^∗^		
	Gene	SD	CV	Gene	SD	CV	Gene	SD	CV	Gene	SD	CV	Gene	SD	CV
1	*Actin*	0.49	2.40	*GAPDH*	0.23	1.18	*GAPDH*	0.32	1.47	*18S*	0.67	3.94	*SAM*	0.39	1.85
2	*SAM*	0.58	2.99	*SAM*	0.30	1.51	*TBP*	0.34	1.41	*SAM*	1.42	5.90	*18S*	0.40	2.58
3	*18S*	0.94	5.95	*TBP*	0.36	1.49	*18S*	0.36	2.58	*Actin*	1.53	6.46	*Actin*	0.51	2.39
4	*GAPDH*	0.96	4.67	*Actin*	0.43	2.12	*SAM*	0.43	2.13	*GAPDH*	1.67	7.14	*TBP*	0.65	2.56
5	*TBP*	1.01	3.96	*18S*	0.65	4.51	*Tubulin*	0.65	3.26	*TBP*	1.69	6.20	*GAPDH*	0.70	3.28
6	*Tubulin*	1.22	6.33	*Tubulin*	0.68	3.77	*Actin*	0.74	3.47	*Tubulin*	2.13	8.95	*Tubulin*	0.86	4.22
7	*MUB*	1.25	5.12	*MUB*	1.41	6.02	*MUB*	1.53	6.37	*MUB*	2.50	9.35	*MUB*	1.61	6.54
8	*HSP90*	3.36	14.52	*HSP90*	2.11	9.65	*HSP90*	2.41	10.71	*HSP90*	2.57	9.91	*HSP90*	2.34	10.02

*^∗^ Total: Pooled samples from all treatments.*

### RefFinder Analysis

The rankings of the four algorithms were integrated by RefFinder to acquire reliable results for the expression stabilities of the eight candidate reference genes of *H. serrata*, and the results are shown in Table 5. The expression of *GAPDH* was ranked as the most stable in the root and leaf, and the expression of *actin* was ranked as the most stable in the stem and sporangia. The expression of *TBP* was ranked the most stable in total. By contrast, *MUB* and *HSP90* were two least stable reference genes almost in all tissues calculated by all five programs. Overall, the best reference genes for accurate transcript normalization in all of the samples were *actin, GAPDH*, and *TBP*, which had the lowest geometric mean of the ranking values.

**Table 5:** Expression stability of candidate reference genes, as assayed with RefFinder software.

Method	1	2	3	4	5	6	7	8
**Ranking Order in Root (Better–Good–Average)**								
Delta CT	*GAPDH*	*TBP*	*SAM*	*Tubulin*	*Actin*	*18S*	*MUB*	*HSP90*
BestKeeper	*Actin*	*SAM*	*18S*	*GAPDH*	*TBP*	*Tubulin*	*MUB*	*HSP90*
NormFinder	*GAPDH*	*TBP*	*Tubulin*	*SAM*	*Actin*	*18S*	*MUB*	*HSP90*
geNorm	*TBP | GAPDH*		*Tubulin*	*SAM*	*Actin*	*18S*	*MUB*	*HSP90*
Recommended comprehensive ranking	*GAPDH*	*TBP*	*SAM*	*Actin*	*Tubulin*	*18S*	*MUB*	*HSP90*
**Ranking Order in Stem (Better–Good–Average)**								
Delta CT	*Actin*	*TBP*	*GAPDH*	*Tubulin*	*18S*	*SAM*	*MUB*	*HSP90*
BestKeeper	*GAPDH*	*SAM*	*TBP*	*Actin*	*18S*	*Tubulin*	*MUB*	*HSP90*
NormFinder	*Actin*	*TBP*	*GAPDH*	*Tubulin*	*SAM*	*18S*	*MUB*	*HSP90*
geNorm	*Actin | TBP*		*GAPDH*	*Tubulin*	*18S*	*SAM*	*MUB*	*HSP90*
Recommended comprehensive ranking	*Actin*	*TBP*	*GAPDH*	*SAM*	*Tubulin*	*18S*	*MUB*	*HSP90*
**Ranking Order in Leaf (Better–Good–Average)**								
Delta CT	*GAPDH*	*TBP*	*SAM*	*18S*	*Actin*	*Tubulin*	*MUB*	*HSP90*
BestKeeper	*GAPDH*	*TBP*	*18S*	*SAM*	*Tubulin*	*Actin*	*MUB*	*HSP90*
NormFinder	*GAPDH*	*TBP*	*18S*	*SAM*	*Actin*	*Tubulin*	*MUB*	*HSP90*
geNorm	*TBP | GAPDH*		*18S*	*SAM*	*Actin*	*Tubulin*	*MUB*	*HSP90*
Recommended comprehensive ranking	*GAPDH*	*TBP*	*18S*	*SAM*	*Actin*	*Tubulin*	*MUB*	*HSP90*
**Ranking Order in Sporangia (Better–Good–Average)**								
Delta CT	*Actin*	*TBP*	*SAM*	*GAPDH*	*Tubulin*	*18S*	*HSP90*	*MUB*
BestKeeper	*18S*	*SAM*	*Actin*	*GAPDH*	*TBP*	*Tubulin*	*MUB*	*HSP90*
NormFinder	*SAM*	*Actin*	*TBP*	*GAPDH*	*Tubulin*	*18S*	*HSP90*	*MUB*
geNorm	*Actin | SAM*		*TBP*	*GAPDH*	*Tubulin*	*HSP90*	*18S*	*MUB*
Recommended comprehensive ranking	*Actin*	*SAM*	*TBP*	*18S*	*GAPDH*	*Tubulin*	*HSP90*	*MUB*
**Ranking Order in Total (Better–Good–Average)**								
Delta CT	*TBP*	*Actin*	*GAPDH*	*SAM*	*18S*	*Tubulin*	*MUB*	*HSP90*
BestKeeper	*SAM*	*18S*	*Actin*	*TBP*	*GAPDH*	*Tubulin*	*MUB*	*HSP90*
NormFinder	*GAPDH*	*TBP*	*18S*	*Actin*	*SAM*	*Tubulin*	*MUB*	*HSP90*
geNorm	*TBP | GAPDH*		*Actin*	*SAM*	*18S*	*Tubulin*	*MUB*	*HSP90*
Recommended comprehensive ranking	*TBP*	*GAPDH*	*Actin*	*SAM*	*18S*	*Tubulin*	*MUB*	*HSP90*

### Validation of the Identified Reference Genes

**FIGURE 8:**
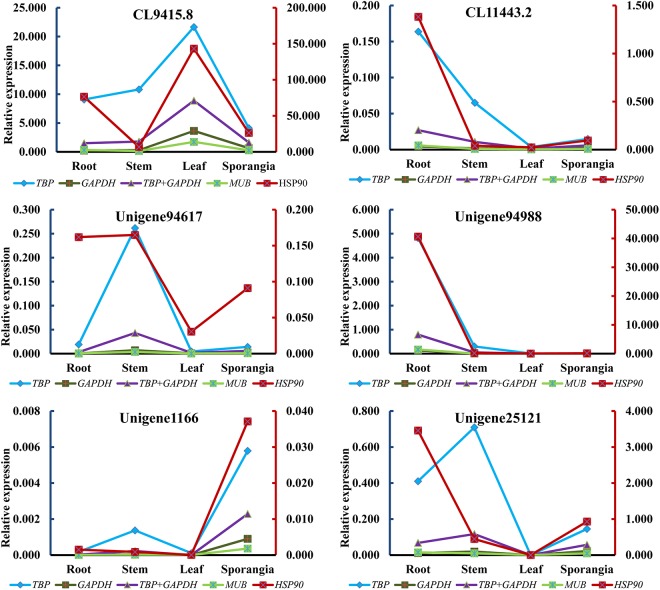
Relative expression patterns of CL94158.8, CL11443.2, Unigene94617, Unigene94988, Unigene1166, and Unigene25121.

The expression levels of HupA biosynthesis-related genes, *L/ODC* (Unigene94617, Unigene94988), and cytochrome P450s (CL94158.8, CL11443.2, Unigene1166, and Unigene25121) were investigated using different reference genes in different tissues at different developmental stages to validate the selected candidate reference genes. Each of the two most stable reference genes (*TBP* and *GAPDH*), its combination (*TBP* + *GAPDH*), and the two least stable reference genes (*HSP90* and *MUB*) were used as internal controls. When using *TBP* alone, *GAPDH* alone, *MUB* alone, and the combination of *TBP* + *GAPDH* for normalization, the expression patterns were similar in all six validated genes. However, when the least stable gene *HSP90* was used for normalization, the expression patterns showed some differences (Figure 8). Thus, RNA Seq-assisted selection of candidate reference genes was helpful.

## Discussion

Standardization and quality assessment of traditional herbal formulations is of paramount importance in order to modernize. However, still major bottlenecks faced by the herbal industry is the unavailability of rigid quality control profiles, primarily because of the complexity and incomplete knowledge of the active medicinal compounds. *H. serrata*, a vulnerable group of slow-growing plant, extensively harvested by the traditional medicinal practitioners. It contains many active compounds, especially HupA whose contents differed significantly among the organs, varieties, age, and production areas of the herbal medicines (Ma et al., 2006). Hence, to address such variation in quality of medicinal material, studies has been directed towards understanding the molecular regulatory mechanisms of secondary metabolism through transcriptomics or functional genomics approaches.

Most of the modern plant research is often underpinned by the genetic approach creating transgenic lines to test the gene functions *in planta*. Inability to genetically transform any lycophytes species such as *H. serrata* has been challenging, and as such our understanding of Huperzia development lags significantly behind almost all other land plant lineages despite its traditional medicinal application. Recently, our group published the global RNA-seq of four different tissues which assisted us for the gene mining regards to the HupA biosynthesi (Yang et al., 2017). Elucidating the biosynthetic pathway is a prerequisite to heterologous production of targeted metabolites limiting the overexploitation of the natural habitat. To our knowledge no suitable reference gene for this plant is available. It is important to select a suitable reference gene to study the different expression patterns in different varieties and different tissues in medicinal plants. Here, we report the use of eight genes (*actin, tubulin, 18S, TBP, GAPDH, HSP90, MUB, and SAM*), to select and validate the suitable reference genes for the qRT-PCR normalization in different tissues and developmental growth stages.

qRT-PCR is one of the most commonly used technologies for transcript expression analysis owing to its sensitivity and reproducibility (Derveaux et al., 2010). Coexpression analysis is a useful method to screen the candidate structure genes involved in specialized metabolite biosynthesis (Saito et al., 2008). Normalization with stable reference genes is critical for obtaining accurate results from qRT-PCR data. Differential and coexpression analyses of the structural genes derived from qRT-PCR have been successful to screen UDP-dependent glucuronosyltransferase, which can catalyze continuous two-step glucuronosylation of glycyrrhetinic acid to yield (Xu et al., 2016). Hence, differential analysis coupled with coexpression analysis will be a useful method to screen the specific genes in the plants without genome information.

For *H. serrata*, the global RNA-seq data from four different tissues have been published, which can be directly used for differential and coexpression analyses. However, the suitable reference gene for this plant is still not selected, which may result in different expression patterns in different tissues or treatments. Here, eight reported reference genes (*actin, tubulin, 18S, TBP, GAPDH, HSP90, MUB*, and *SAM*) were selected and validated to discover the suitable reference genes for the qRT-PCR normalization in different tissues.

In the current study, four housekeeping genes (*GAPDH, actin, tubulin*, and *18S*) and other four genes (*TBP, SAM, HSP90*, and *MUB*) were used as query genes for the blast against the RNA-seq data (Yang et al., 2017) to find the homologous genes (Stanton et al., 2017). Genes with similar expression levels in four different tissues were selected as candidates as previously reported (Tan et al., 2015). In this study, the traditional reference genes, *GAPDH, actin, tubulin*, and *18S*, had good performances in CV values in qRT-PCR Cq values (CV < 10%), in line with the previous reports (Shivhare and Lata, 2016; Martins et al., 2017). The four most extensively used programs (ΔCq, geNorm, NormFinder, and BestKeeper) were used in this study for analyzing the stabilities of candidate reference genes to avoid selection of coregulated genes. The four programs showed a few differences in results; *TBP* and *GAPDH* were the most two stable reference genes, *MUB* and *HSP90* were the least stable reference genes, and others were midstable candidates with different rankings calculated by different programs.

Although *TBP* was the most stable candidate for all samples in our study, its expression level was very low, which was also observed in equine milk somatic cells and in *Aedes aegypti* (Cieslak et al., 2015; Dzaki et al., 2017). This low level was due to that the Cq values in qRT-PCR assays varied from 21.34 to 31.05 in all experiments with the five dilution cDNAs, which indicated that the *TBP* is a new plant species-dependent reference gene, hence suggesting a proper validation in each case. While, *GAPDH* exhibited good performance on qRT-PCR normalization in different tissues of plants of different developmental stages, as calculated by most of the programs. Hence, *GAPDH* alone is suitable for qRT-PCR normalization as a reference gene under different tissues. The few differences in reference genes showed in different programs were also in agreement with earlier studies (Jain et al., 2006; Cruz et al., 2009; Jain, 2009; Qi et al., 2016). The different rankings of the reference genes showed in different programs were also observed in *chrysanthemum* (Gu et al., 2011; Wang et al., 2015); thus, all these programs must be combined to evaluate the candidates for each species.

We further performed RT-qPCR experiments to investigate the expression levels of *L/ODC* genes, which were previously characterized in *H. serrata, Lycopodium clavatum*, and Leguminosae (Bunsupa et al., 2012; Bunsupa et al., 2016; Xu et al., 2017) by using the two most stable reference genes (*TBP* and *GAPDH*) and the two least stable reference genes (*MUB* and *HSP90*), to evaluate the eight selected reference genes. According to the pairwise analysis by geNorm software, two reference genes were sufficient for the normalization; thus, the combination of *TBP* and *GAPDH* was also used to calculate the expression level of targeted genes. Regardless of which reference gene was used, the expression patterns of *L/ODC* (Unigene94988) were the same. To further validate, Unigene94617, a homologous gene of Unigene94988, and four cytochrome P450 genes (CL9415.8, CL1143.2, Unigene1166, and Unigene25121) proposed to participate in HupA biosynthesis (Yang et al., 2017) were employed for the normalization. All reference genes, with exception of *HSP90*, acquired a similar expression pattern for all targeted genes. Hence, only *HSP90* was unsuitable for qRT-PCR normalization in all tissues of *H. serrata*, which suggested that RNA-seq-assisted selection was a useful method for selecting suitable reference genes. Previous studies in *Arabidopsis thaliana, Coffeea arabica, Gossypium hirsutum*, and *Chrysanthemum* showed that the novel reference genes exhibited better performance than traditional reference genes (Czechowski et al., 2005; Cruz et al., 2009; Artico et al., 2010; Qi et al., 2016). Taken all together, although we observed some inconsistency on the expression patterns of the some genes in HupA biosynthesis between RNA-seq and qRT-PCR, this might be due to the plant growth condition differences (season and climate) when we collected (Supplementary Table S1). The major reason for this is likely due to the seasonal and climatic factors or growth as this plant takes years to grow (Ma et al., 2006). Similarly, inconsistency was also observed previous reports. In many cases, the gene expressions quantified with different methods were dramatically different (Wang et al., 2006; Marioni et al., 2008; Qin et al., 2013; Rajkumar et al., 2015; Dapas et al., 2017). Due to the lack of successful *in vitro* propagation approach of Lycopodiaceae family, its important to design such functional genomics study from the control climatic conditions and/or established *in vitro* platform. Our lab is currently exploring the approach of *in vitro* propagation of endangered species of Lycopodiaceae family. This study state possible use of housekeeping genes as a stable candidate for qRT-PCR normalization of plants belonging to Lycopodiaceaea family especially *H. serrata*.

## Conclusion

In this study, we proposed *H. serrata* as a model plant for functional genomics study in the Lycopodiaceae family. The qRT-PCR reference gene normalization in tissues of *H. serrata* showed that *TBP* and *GAPDH* were the two most suitable reference genes. The combination of the two genes as reference genes was accurate for qRT-PCR normalization, as performed in different tissues of *H. serrata* according to the pairwise variation analysis by geNorm program. The reference genes identified and validated here through RNA-seq data for qRT-PCR normalization will facilitate the establishment of standardized qRT-PCR program for other genetically close plants.

## Author Contributions

YX conceived the research. YX and SW designed the experiments. WY, SW, and MY, performed the experiments. MY and SW analyzed the data, wrote the manuscript, and coordinated its revision. YX and AJ revised the manuscript. All authors provided helpful discussions and approved the final version.

## Conflict of Interest Statement

The authors declare that the research was conducted in the absence of any commercial or financial relationships that could be construed as a potential conflict of interest.

## Footnotes

1 http://www.theplantlist.org/tpl1.1/record/tro-26621719

2 http://www.cvh.ac.cn/en/spm/HUST/00019690

## Supplementary Material

TABLE S1 The consistency of target genes expression and the expression data of qRT-PCR and RNA-seq.
